# Robotic renal cyst decortication with calyceal diverticulectomy in a toddler – technical practicalities: a case report

**DOI:** 10.1186/s13256-018-1830-9

**Published:** 2018-10-02

**Authors:** Yi-Chun Wang, Jia-Dong Xia, Qi-Jie Zhang, Chen Chen, Jian-Xin Xue, Jie Yang, Chao Qin, Ning-Hong Song, Zeng-Jun Wang

**Affiliations:** Department of Urology, The First Affiliated Hospital of Nanjing Medical University, 300 Guangzhou Road, Nanjing, China

**Keywords:** Robot assisted, Renal cyst, Calyceal diverticula, Pediatric

## Abstract

**Background:**

Incidence of simultaneous renal cyst with calyceal diverticula in contralateral kidney is rare in children. A minimally invasive procedure in different sittings is often recommended.

**Case presentation:**

A Chinese 15-month-old boy presented to the Urology department of a tertiary care center with right flank pain. He was subjected to magnetic resonance urography and was diagnosed as having right renal cyst and contralateral calyceal diverticula. He underwent robotic cyst decortication and calyceal diverticulectomy using da Vinci robot. His postoperative period was uneventful. He was discharged on fifth postoperative day. Histopathology was consistent with simple renal cyst.

**Conclusions:**

Robotic combined cyst decortication and contralateral diverticulectomy is feasible in selected small children. However, it demands adequate technical skill and experience.

## Background

A calyceal diverticulum is an outpouching of the renal collecting system lying within the renal parenchyma [1]. It is lined by transitional cell epithelium and connects with the collecting system via a narrow channel, which results in passive filling of urine. In the long term, it can lead to the formation of stone, infection, and other symptoms including pain and increasing size, which need surgical intervention. The exact etiology of calyceal diverticulum is still unknown. However, the hypothesis about congenital origins is highly regarded [2].

A simple renal cyst (SRC) is defined as a solitary discrete lesion outside the kidney parenchyma [3]. It has been reported that the prevalence of SRC ranges from 5 to 41% in adults; the rate in children is low but it increases with age from 0.22 to 0.55% [4]. Most SRCs are asymptomatic and discovered incidentally. However, some large SRCs can occasionally cause some symptoms like flank pain.

Robot-assisted surgery with the da Vinci Surgical System is increasingly being applied in a wide range of surgical specialties, especially in urology [5]. It overcomes the limitations of the standard laparoscopic approach and allows for precise dissection in a confined space. However, there are few articles reporting the procedure of robot-assisted one-stage surgery for bilateral renal lesions, especially in toddlers. To show the feasibility of this technique and critically evaluate it, we reported this case of robot-assisted simultaneous cyst decortication and calyceal diverticulectomy.

## Case presentation

A Chinese 15-month-old boy presented to the Urology department of a tertiary care center with right flank pain. He had no hypertension or fever. In addition, there were no urinary symptoms such as crying or straining during micturition, frequent urination, or hematuria for the child. According to the history taken from his parents, the pregnancy was uneventful, with no significant antenatal and postnatal history. His birth weight was 2.9 kg, height 51 cm, and head circumference 36 cm. Past medical, environmental, and family history were also not significant. There were no congenital anomalies in the family. He had no siblings. On examination, his pulse rate was 115 beats per minute, body temperature 36.7 °C, respiration rate 28 breaths per minute, and blood pressure 95/46 mmHg. There was no tenderness and no mass palpable in his flank.

**Fig. 1:**
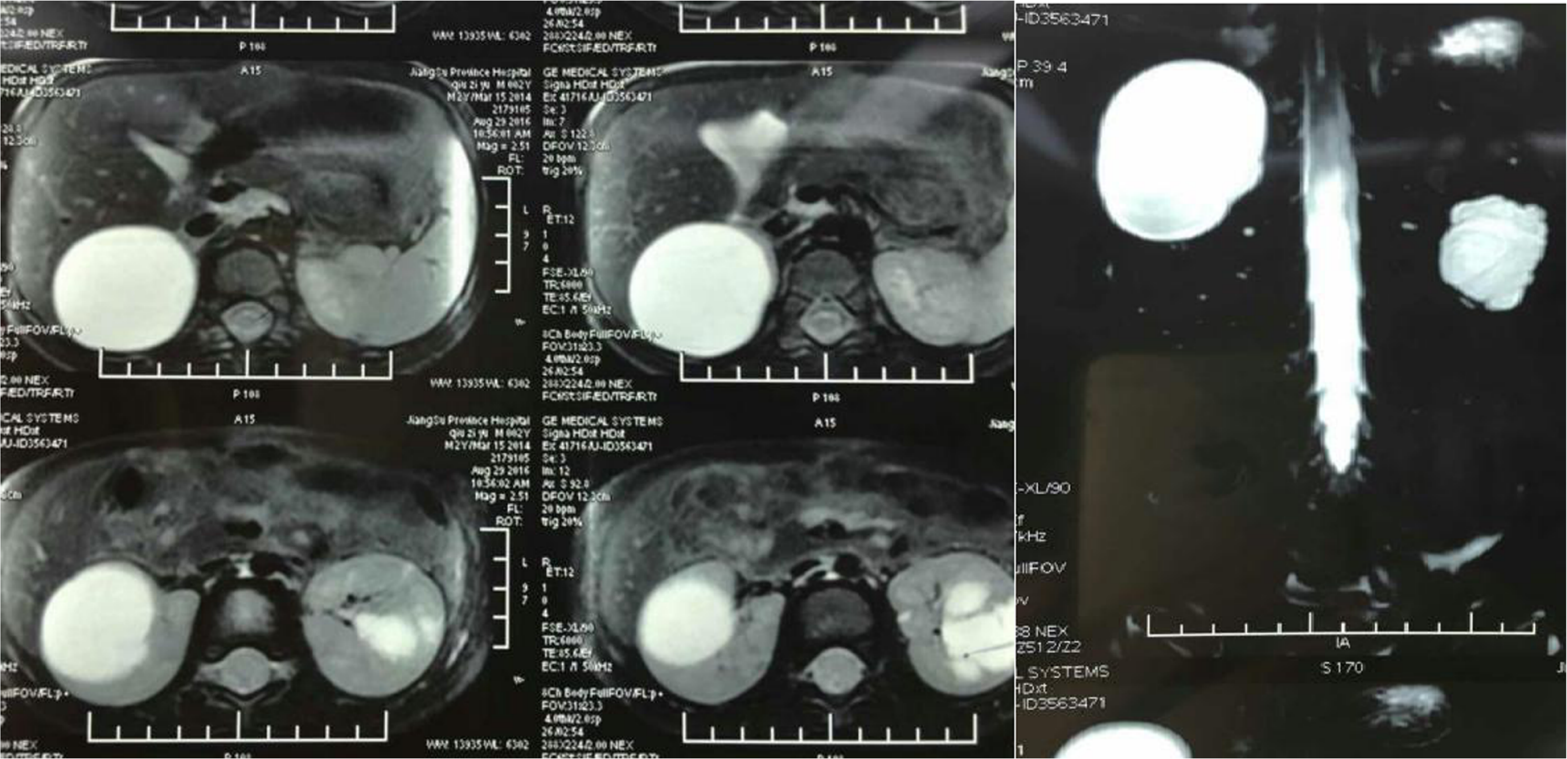
Magnetic resonance urography shows: a thin-wall renal cyst measuring 60 mm (maximum diameter) in the upper part of right kidney and a multilocular cystic lesion (approximately 40 mm) in the bottom of left kidney

**Fig. 2:**
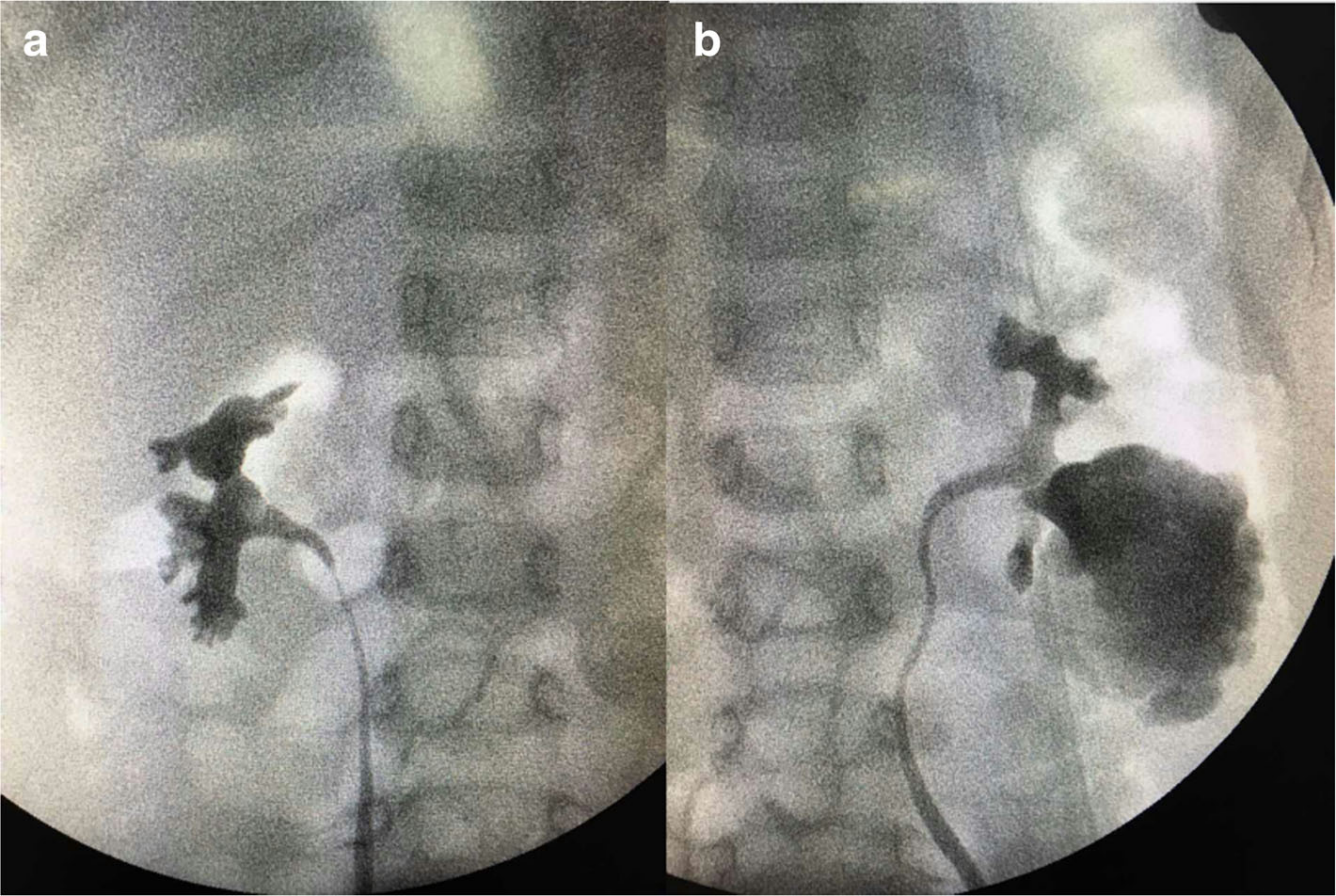
Retrograde pyelography shows: **a** the cyst not communicating with the collecting system; **b** the cystic area communicating with the collecting system via a narrow infundibulum

The abdominal ultrasound from the local hospital revealed multiple cystic lesions in both kidneys. Magnetic resonance urography (MRU) indicated the possibility of a renal cyst (right; Fig. 1) and calyceal diverticula (left); a parapelvic cyst was not excluded. For further evaluation, retrograde pyelography was done during an operation (Fig. 2). At last, the child was reliably diagnosed as having right renal cyst and calyceal diverticula on the other side. No significant abnormality was found in the results of laboratory findings, including complete blood count (CBC), liver and renal functions, urine analysis, serology, and microbiology. (Table 1).

**Table 1:** Results of laboratory findings

	Result(s)	Unit(s)	Range(s) of normal value
WBC	9.40	10^9^/L	3.50–9.50
RBC	4.54	10^12^/L	4.30–5.80
PLT	121↓	10^9^/L	125–350
Hb	126↓	g/L	130–175
ALT	15.3	U/L	9–50
AST	29.5	U/L	15–40
TBIL	11.3	umol/L	5.1–19.0
DBIL	3.3	umol/L	0.0–6.8
IBIL	8.0	umol/L	0.0–20.0
TC	5.21	mmol/L	0.00–6.20
TG	0.81	mmol/L	0.00–2.25
ALB	47.8	g/L	40.0–55.0
GLU	5.50	mmol/L	3.90–6.10
Urea	4.20	mmol/L	2.90–8.20
Cr	26.5↓	umol/L	44.0–133.0
UA	276.9	umol/L	208–428
PT	12.20	second	11 ± 3
APTT	25.00	second	24.5 ± 10

*ALB* albumin, *ALT* alanine aminotransferase, *APTT* activated partial thromboplastin time, *AST* aspartate aminotransferase, *Cr* creatinine, *DBIL* direct bilirubin, *GLU* glucose, *Hb* hemoglobin, *IBIL* indirect bilirubin, *PLT* platelets, *PT* prothrombin time, *RBC* red blood cells, *TBIL* total bilirubin, *TC* total cholesterol, *TG* triglyceride level, *UA* uric acid, *WBC* white blood cells

**Fig. 3:**
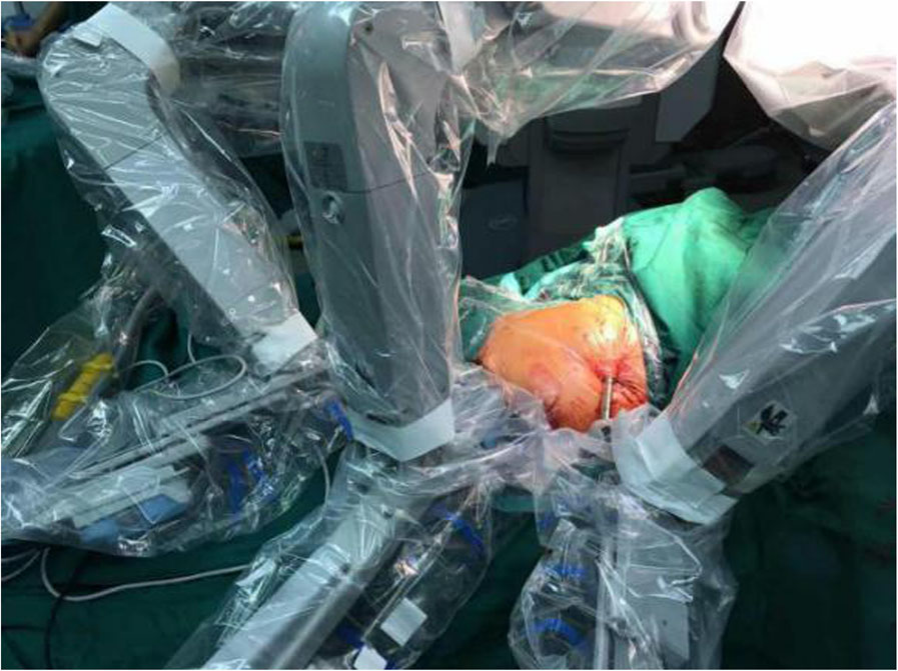
Port placement

**Fig. 4:**
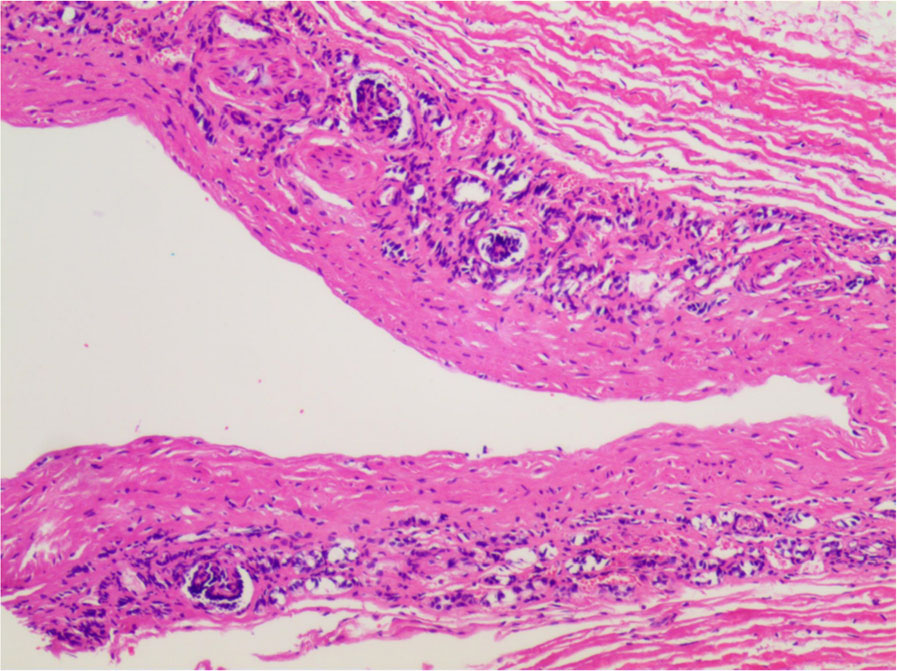
Microscopic picture shows simple renal cyst

A robotic-assisted procedure was planned. On September 1, 2016, the procedure was performed using a three-arm da Vinci Robot, standard version, starting from the right side. Our patient was secured in a flank position with the table slightly bent. Regarding the port placement, five ports in the abdomen region were placed for the procedure, with a 12.0 mm camera port, two 8.0 mm robotic ports, and a 12.0 mm and 5.0 mm assistant port (Fig. 3). Subsequently, the robot was docked over the shoulder with the camera oriented in accordance with the kidney. The robotic grasper in Arm 1 for retraction and monopolar scissors in Arm 2 were used under the camera guidance with 30-degree down-scope. The ascending colon was mobilized and reflected medially to expose the kidney. After carefully clearing away the perinephric fat to expose the cyst, it was incised and fluid was carefully aspirated. Most of the cyst wall was excised and sent for histopathological examination (Fig. 4). This was followed by contralateral procedure on right side in same sitting. The calyceal diverticula was found using the same previously reported procedures and unroofed completely without renal arterial clamping. Renorrhaphy was performed in two layers using sliding-clip technique. Surgical drains were placed on both sides. Operating time was 90 minutes. Postoperative drainage from two drains was approximately 50 cc. No prophylactic antibiotics were given before surgery. Intravenously administered cefodizime (1 g, twice a day) was given 48 hours after surgery for prophylactic purpose. His postoperative period was uneventful and he was discharged on day five postoperatively. With 1-year follow up after the surgery, the child did not have any complaints, and postoperative ultrasound images showed no hydrops or diverticulum associated with either kidney.

## Discussion

In this case, robotic renal cyst decortication with calyceal diverticulectomy was performed successfully in a toddler. As we know, few similar cases were reported due to low incidence rate in children and immature technology. Therefore, this case report helped to show the feasibility and safety of this technique for children.

Calyceal diverticula are rarely diagnosed in children [6–10]. They are most often congenital; they are rarely acquired after urinary tract infection, trauma, or urolithiasis. They constitute a diagnostic challenge in children due to the ultrasonographic resemblance to solitary renal cysts, which are also rare in this age group [11]. In this case, we took retrograde urography to distinguish calyceal diverticula from renal cyst during the operation, which is effective and reliable. Although the diverticulum of the left kidney was asymptomatic, it is necessary to deal with the lesions on both kidneys simultaneously. In the long term, calyceal diverticulum can lead to the formation of stone, infection, and other symptoms, which need surgical intervention. Previous research has established that stones can be found in up to 50% of calyceal diverticula, although, over the combined reported series, 96% of patients presented with stones [12]. So we performed calyceal diverticulectomy to reduce the risk of more aggressive operations in the future, which is more beneficial to the patient.

Compared with staging operation, the risk of one-stage surgery for bilateral renal lesions is much higher. Soylu Boy *et al*. [13] reported two cases of bilateral synchronous sporadic renal cell carcinoma (RCC), which were successfully treated by laparoscopic partial nephrectomy. It is further indicated that simultaneous bilateral renal surgery is safe and feasible, and the advantages are obvious: it avoids the pain of secondary surgery, shortens the hospital stay, and saves medical expenses. However, not all patients are suitable for the treatment of bilateral lesions at the same time. It is a demanding job to select appropriate patients and analyze the examination results: (1) To avoid unnecessary excessive separation and damage, it is important to determine the location, size, number, and neighboring relationship of the lesions via imaging examination. (2) For bilateral kidney diseases, the surgical procedure should give priority to the easy-to-operate side, and then do the other side, so that at least one side of the surgery can be guaranteed. If there are some difficulties or accidents, the latter operation can be terminated or changed to open surgery at any time depending on the situation. In this case, the right renal cyst is easier to treat than the left renal diverticulum, so the right-side surgery is performed first. (3) Some easily overlooked surgical contraindications should be removed when selecting patients, such as history of abdominal surgery, obesity, chronic obstructive pulmonary disease in patients with hypercapnia, and poor physical condition (old, poor liver and kidney function). (4) Compared to unilateral surgery, the risk of anesthesia, hypercapnia, subcutaneous emphysema, and other laparoscopic-related complications could increase due to extended operation time.

The management of calyceal diverticula has been debated. Laparoscopy is an alternative approach that has been used with favorable results [14]. Compared with traditional laparoscopic surgery, robotic surgery is featured with a wrist function at the tip, movement downgrading, tremor elimination, a stable three-dimensional vision, greater precision in dissection, easier suturing and knot tying, shorter learning curve, and favorable surgical ergonomics. It may help the surgeon overcome some of difficulties encountered with traditional laparoscopic surgery and have the potential to reduce intraoperative blood loss, perioperative complications, and length of hospital stay [15, 16]. In 2007, Driscoll and Kim [17] reported a robot-assisted laparoscopic calyceal diverticulectomy in a 14-year-old girl, which was performed successfully. Considering the age and narrow operation space, we also decided to perform robot-assisted cyst decortication and calyceal diverticulectomy simultaneously, which has been rarely reported before. Although the robot-assisted intervention was not less cost effective than laparoscopic surgery, our patient’s posture had to be switched to fit the need of different kidney surgeries. In fact, the two surgeries were performed without shutting the robot down and using the same surgical instruments. These aspects helped strongly to decrease the costs of a robotic-assisted operation. Overall, the entire operation lasted approximately 90 minutes including anesthesiological procedures, patient positioning, and trocar placement. We think that this is acceptable for a bilateral procedure as confirmed by the regular observations made in the postoperative period.

However, some limitations of our technique exist. As mentioned above, it is a demanding job to select suitable patients. Some easily overlooked surgical contraindications should also be removed. Moreover, laparoscopic experience is indispensable for surgeons to shorten the operation time and reduce the risk of laparoscopic-related complications as much as possible.

## Conclusions

Our experience was encouraging and confirmed the feasibility and the safety of this procedure for children. Robot-assisted laparoscopy is a useful tool with excellent visualization and precise maneuvering. However, an appropriate selection of the patients and a skill in robot-assisted renal surgery are mandatory before approaching this type of surgery.
